# Spanish Adaptation of the Dimensional Apathy Scale (DAS) in Amyotrophic Lateral Sclerosis

**DOI:** 10.3389/fneur.2020.562837

**Published:** 2020-10-06

**Authors:** Teresa Salas, Ratko Radakovic, Víctor Rodriguez-Castillo, Saúl Marín, Delia Chaverri, Francisco Rodriguez-Santos

**Affiliations:** ^1^Neurology Service, Hospital Universitario La Paz, Madrid, Spain; ^2^Faculty of Medicine and Health Sciences, University of East Anglia, Norwich, United Kingdom; ^3^Norfolk and Norwich University Hospital, Norwich, United Kingdom; ^4^The Euan MacDonald Centre for Motor Neurone Disease, University of Edinburgh, Edinburgh, United Kingdom; ^5^Alzheimer Scotland Dementia Research Centre, University of Edinburgh, Edinburgh, United Kingdom; ^6^Centre for Cognitive Ageing and Cognitive Epidemiology, University of Edinburgh, Edinburgh, United Kingdom; ^7^Neuropsychology Unit, UDEN-GiuntiEOS, Madrid, Spain

**Keywords:** apathy, validity, reliability, amyotrophic lateral sclerosis, translation, Spanish version

## Abstract

**Aim:** To adapt, translate, and utilize the Dimensional Apathy Scale (DAS) in Amyotrophic Lateral Sclerosis (ALS) to the Spanish population.

**Method:** We recruited 104 ALS patients (67 of their caregivers) and 49 controls. Participants completed the Spanish-translated DAS, Geriatric Depression Scale- Short form. Patients were also administered the ALS Functional Rating Scale-Revised (ALSFRS-R). Caregivers additionally completed the informant/caregiver-rated Spanish-translated DAS. The DAS was translated to Spanish using a back-translation method. Test-retest and internal consistency reliability were examined. Divergent validity was assessed by comparing the DAS with the depression scale (GDS-15). Principal Component Analysis (PCA) was applied to explore the substructure of the Spanish DAS.

**Results:** The internal consistency reliability of self-rated Spanish DAS was 0.72 and of the informant/caregiver-rated Spanish DAS was 0.84. Correlations between self-rated DAS subscales and GDS-15 were not statistically significant, with a good test-retest reliability. PCA analysis showed a similar substructure to the original DAS. ALS patients had significantly higher Initiation apathy than controls. Additionally, ALS patient informant/caregiver-rated DAS Emotional apathy was significantly higher than the self-rated, with no significant differences observed in the Executive and Initiation subscales. No association was found between DAS and functional impairment using the ALS Functional Rating Scale (ALSFRS-R).

**Conclusion:** The Spanish translation of the DAS is valid and reliable for use in assessing multidimensional apathy in the Spanish population. Availability of the Spanish DAS will allow for future research to explore different apathy subtypes and their impact in ALS and other conditions.

## Introduction

Many studies show that apathy is the most common and relevant behavioral symptom in patients with Amyotrophic Lateral Sclerosis (ALS) (1), and it's estimated to be present in 31–56% of patients (2–5). Apathy has been linked to cognitive impairment, as well as to worse prognosis in ALS patients (6).

This symptom is measured using tests that do not take into consideration the physical impairment of patients, such as the Frontal Systems Behavior Scale (FrSBe) (7), or it is assessed globally as part of other behavioral and psychiatric disorders as is the case of the Cambridge Behavioral Inventory revised (CBI-R) (8), the ALS Frontotemporal Dementia Questionnaire (ALS-FTD-Q) (9), or, lastly, the Motor Neuron Disease Behavioral Instrument (MiND-B) (10). However, apathy is established as a multidimensional construct, composed of different subtypes or domains with different cognitive and neurobiological substrates [e.g., (11, 12)]. Apathy as a syndrome has been further recognized in terms of assessment, diagnosis, and management (13–15).

The Dimensional Apathy Scale (DAS) (13) was developed to measure different subtypes of apathy, accounting for physical disability. The DAS quantifies three subtypes of apathy, executive apathy (lack of motivation for planning, organization, and attention); emotional apathy (emotional flatness, blunting, indifference, or neutrality); and initiation apathy (lack of motivation for self-generation of thoughts and/or actions). Initiation apathy, independent of physical disability, has been observed to be characteristic in ALS (16, 17) and has been associated with characteristic cognitive deficits (18). Comparatively different from profiles manifested by other neurodegenerative diseases such as Alzheimer's disease and Parkinson's disease (12). The DAS is a fast, simple, and easily understandable measure of multidimensional apathy that can be used both in the clinical context and in research. Thus, far the DAS has been validated in Italian, French, and Japanese (19–21), with no Spanish translation available.

The aim of this study was to validate the translation of DAS to the Spanish population. Additionally, the aim was to explore the profile of apathy in Spanish ALS patients.

## Methods and Materials

### Participants

We recruited 104 ALS patients (61 male and 43 female), as well as 67 of their caregivers, using a convenience sampling method *via* the ALS Unit at the La Paz-Carlos III Hospital, Madrid, Spain, a multidisciplinary care and research facility. All patients were diagnosed with probable or definite ALS (22). The exclusion criteria for patients were tracheostomy or use of noninvasive ventilation for more than 2 h during waking hours at the time of the study, other neurological impairment, brain injury, or psychiatric illness and history of substance abuse. In all, 11 ALS patients declined to take part in the research, and one passed away during the study.

Caregivers must provide regular care and have substantial contact with the patient participant. The caregivers had a minimum of 10 h total and at least 3 days contact per week with the patient participant.

We also recruited 49 controls (21 male and 28 female) using a convenience sampling method and included friends, family, salaried caregivers, or spouses of patients. The exclusion criteria for controls were a prior clinical history of neurological impairment/dysfunction or psychiatric illness.

### Procedures and Measures

Both the patient and the control groups provided demographics information and completed self-rated measures of apathy (Dimensional Apathy Scale) and depression (Geriatric Depression Scale- Short Form), as well as a measure of functional disability (ALS Functional Rating Scale-Revised) and a cognitive screen (Edinburgh Cognitive and Behavioral ALS Screen). All measures could be completed either by writing or verbally, to allow for varying physical disability and disease stages associated with ALS. The caregiver group informant-caregiver-rated versions of the Dimensional Apathy Scale (Informant/caregiver-rated versions), as well as a behavioral interview (Edinburgh Cognitive and Behavioral ALS Screen), about their observations of the patients. Please see below for descriptions of the measures.

The Dimensional Apathy Scale [DAS; (16, 23)] is composed of three subscales assessing different dimensions of apathy: Executive, Emotional, and Initiation. Each item is answered on a 4-point Likert scale of frequency in the last month. The maximum score for each subscale ranges from 0 (no apathy) to 24 (most apathy). Clinical cutoffs were available: executive apathy ≥14, emotional apathy ≥15, and initiation apathy ≥16 (16). Both self-rated and informant/caregiver-rated versions are available and were translated into Spanish using a back-translation method by a professional bilingual translator. This was then reviewed by two ALS experts before finalization. Please see Supplementary Material for the Spanish DAS (self-rated and informant/caregiver-rated versions) and scoring sheets.

The Geriatric Depression Scale-Short Form [GDS-15; (24)] is composed of 15 items answered dichotomously (Yes/No) based on the occurrence of depressive symptoms during the previous week. The total score ranges from 0 (no depressive symptoms) to 15 (high depressive symptoms), with the cutoff score set at 5. A Spanish translation was available (25).

The Edinburgh Cognitive and Behavioral ALS Screen [ECAS; (26)] assesses cognitive functions (executive functions, social cognition, verbal fluency, language, memory, and visuospatial function), producing a score ranging from 0 to 136, with lower scores indicating more cognitive impairment. The ECAS also contains a behavioral interview to be completed by the caregiver about the patient to determine presence of behavioral symptoms (e.g., disinhibition, apathy, loss of sympathy and empathy, perseverative/ritualistic behavior, altered food preference, and psychosis). A Spanish translation was available (27).

The ALS Functional Rating Scale-Revised [ALSFRS-R; (28)]: ALSFRS-R is a tool designed to measure disease progress in ALS patients. It consists of 12 items, each scored on a 5-point Likert scale assessing functional disability, with a total score ranging from 0 (least functional ability) to 48 (most functional ability). A Spanish translation was available (29).

The study received institutional ethical approval by the researchers at Institute University Hospital La Paz (IdiPAZ). All patient, control, and caregiver participants gave informed consent following the Declaration of Helsinki.

### Statistical Analysis

R software was used for statistical analysis. The Shapiro-Wilk test was used as a test of normality to determine whether the analysis should be parametric or nonparametric. Demographic variables comparison between ALS patients and controls was assessed through *t*-tests (age) and chi-square tests (sex and education level). Internal consistency reliability was assessed using Cronbach's standardized alpha. Intraclass correlation (ICC) was used in order to determine the self-rated DAS test-retest reliability in the patient group in a 1-month interval. Spearman's correlation coefficient (Holm corrected) was used to examine correlations between DAS subscales, DAS Total and with GDS-15. The Mann Whitney U test was used to distinguish, on the DAS Total score, between individuals with apathy and those without (according to the ECAS behavior interview, apathy/inertia domain).

To determine the number of factors extracted based on own values, Horn's parallel analysis of principal factors was used. To determine the substructure of the scale, a Principal Components Analysis (PCA) was performed on the self-rated DAS in Spanish. Derivative factors overlapping and the original DAS subscales were analyzed (% mapped). The scores for the PCA's derived factors were calculated, and in order to determine overlapping, Spearman's correlation coefficient was applied using the original scores for the self-rated Spanish DAS.

The ALS patients' DAS scores were compared to the caregivers scores using *t*-tests. For the control comparison, the ALS patients were subsampled to match the control group for age, sex, and education, utilizing previously used subsampling methodology (16). To further determine the apathy profile, DAS scores of the ALS subsample group and control group were compared using *t*-tests.

## Results

### Demographic and Clinical Variables

Table 1 shows demographic and clinical descriptors of ALS patients and controls. ALS patients were significantly older than controls. There was no significant difference between ALS patients and controls on sex, education, and depression. It was found that 30.2% of ALS patients were above the cutoff for depression, compared with 22.5% of controls. Further, the most common relationship to the caregiver was that of spouse (*n* = 48), followed by other relatives (*n* = 15), and finally by friends, formal caregivers, and other relationships (*n* = 4).

**Table 1:** Demographic and clinical data of ALS patients and controls.

	**ALS patients (*n* = 104)**	**Control (*n* = 49)**	**Statistics**	***p*-value**
Age (mean ± SD)	59.9 ± 11.6	53.2 ± 10.5	*t* = 3.52	<0.001
Sex (male/female)	61/43	21/28	χ^2^ = 3.34	n.s.
**Education**				
Without university degree % (*n*)	59.6 (62)	51.0 (25)	χ^2^ = 2.45	n.s.
With university degree % (*n*)	32.7 (34)	44.9 (22)		
Unknown % (*n*)	7.7 (8)	4.1 (2)		
GDS-15 (mean ± SD)	3.9 (3.1)^††^	3.1 (2.8)	T = −1.40	n.s.
Duration of symptoms, months	23.31 (29.63)			
Median, IQR	15 (15)			
**Onset region**				
Spinal % (*n*)	68.2 (71)			
Bulbar % (*n*)	30.8 (32)			
Respiratory % (*n*)	1 (1)			
ALSFRS-R (mean ± SD)/48	32.2 ± 8.5^†^			
Cognitive ECAS, Total (mean ± SD)/136	104.5 ± 21.7^‡^			
Behavioral ECAS (median, IQR)/5	0 (1)^‡‡^			

† *n = 96*;

†† *n = 96*;

‡ *n = 55*;

‡‡ *n = 56*.

### Reliability, Validity, and Substructure

Cronbach's standardized alpha was 0.72 for the self-rated Spanish DAS and 0.84 for the informant/caregiver-rated Spanish DAS, pointing to a high internal consistency. The reliability test-retest for the patient group (*n* = 75) for the self-rated DAS subscales was excellent (ICC Executive = 0.85, ICC Emotional = 0.75, ICC Initiation = 0.87). The median of the number of days between test and retest was 30 (IQR = 73).

The correlation between the self-rated DAS subscales showed a similar pattern than that of the original DAS (23), with significant correlations between Initiation and Executive apathy, but these correlations were not observed between Executive and Emotional apathy (Table 2). In terms of divergent validity, neither the self-rated and informant/caregiver-rated DAS subscales nor the DAS total score showed a significant correlation with the depression scale (GDS-15), as shown in Table 2. Further, the informant/caregiver-rated DAS showed significant correlations were observed between the Initiation and Executive subscales, as well as for Initiation and Emotional (see Table 2). No significant correlation was found when comparing the self-rated and informant/caregiver-rated DAS with the ALSFRS-R.

**Table 2:** Correlation between DAS subscales (self-rated and informant carer rated) and GDS-15 for patients.

**Self-rated (*n* = 104)**	**DAS Executive**	**DAS Emotional**	**DAS Initiation**	**DAS Total**	**Self-rated GDS-15**
DAS Executive	1.000	0.097	0.373^**^	0.704^***^	0.064^†^
DAS Emotional		1.000	0.189	0.480^***^	−0.038^†^
DAS Initiation			1.000	0.814^***^	0.128^†^
DAS Total				1.000	0.096^†^
**Informant/caregiver-rated (*****n*** **=** **67)**	**DAS Executive**	**DAS Emotional**	**DAS Initiation**	**DAS Total**	**Self-rated GDS-15**
DAS Executive	1.000	0.189	0.579^***^	0.720^***^	0.276^‡^
DAS Emotional		1.000	0.415^**^	0.657^***^	0.047^‡^
DAS Initiation			1.000	0.657^***^	0.248^‡^
DAS Total				1.000	0.230^‡^

† *n = 86*,

‡ *n = 66*;

* *p < 0.05*;

** *p < 0.01*;

*** *p < 0.001*.

Further, a subset of ALS patients and their caregivers (*n* = 39) completed both the ECAS behavioral interview and the informant/caregiver-rated DAS. Based on the ECAS behavioral interview, patients with apathy/inertia (*n* = 8) had a significantly higher (*U* = 65, *p* < 0.05) informant/caregiver-rated DAS Total score (Median = 31.5) than those without apathy/inertia (Median = 24), showing good convergent validity. No such difference was observed with the self-rated DAS.

A PCA with a Varimax rotation (orthogonal) was used, since the factors were not correlated. Horn's parallel analysis determined that three factors should be extracted. Kaiser-Meyer-Olkin's test (KMO) and the mean Measure of Sampling Adequacy (MSA) showed that the sample can be factored (KMO = 0.665, mean MSA = 0.638). This was backed by a significant value for Bartlett's test of sphericity (*p* < 0.001). The cumulative three factors solution represented 36.1% of total variance and the factors were subsequently labeled according to the content of their items (Table 3). In general, 79% of the items saliently loaded to the original item 24 from DAS. In terms of the AES subscales, 87.5% of the items were correctly assigned to the Initiation and Executive subscales, with 37.5% of the items in the Emotional subscale. Additionally, correlation analysis showed significant positive correlations between self-rated DAS subscales and the corresponding PCA derived scores.

**Table 3:** Orthogonal rotation PCA (principal components analysis) for self-rated Spanish DAS item responses (*n* 104), mapping, and correlation of principal components to original DAS in ALS patients.

	**PC 1 (Initiation)**	**PC 2 (Executive)**	**PC 3 (Emotional)**
D13BCI	**0.770**	−0.177	0.005
D4BCI	**0.725**	0.202	−0.045
D14BCI	**0.592**	0.116	−0.212
D16BCI	**0.575**	−0.127	0.142
D18BCI	**0.553**	**0.339**	0.239
D8BCI	**0.490**	0.035	0.291
D2BCI	**0.413**	0.144	0.158
D19Ex	−0.231	**0.682**	−0.01
D6Ex	0.078	**0.681**	0.030
D21Ex	0.108	**0.673**	**0.391**
D23Ex	−0.026	**0.583**	0.128
D17Ex	0.237	**0.520**	−0.105
D11Ex	**0.610**	**0.448**	−0.079
D1Ex	0.253	**0.470**	−0.042
D5Em	0.058	−0.043	**0.684**
D7Em	0.226	−0.227	**0.550**
D24Em	0.015	0.115	**0.403**
D3Em	**0.534**	0.005	−0.464
D10Ex	**0.354**	0.271	**0.436**
D12Em	0.121	0.158	0.056
D9Em	−0.007	−0.120	0.154
D15Em	−0.003	0.298	−0.135
D20Em	−0.233	−0.016	0.033
D22BCI	0.234	−0.121	−0.502
Proportion of variance	0.153	0.122	0.086
Cumulative variance	0.153	0.275	0.361
% Mapping of original DAS subtypes (loadings ≥ 0.30)	87.5	87.5	37.5
Correlations of PC-derived subscale scores to self-rated DAS	PC 1 (Initiation)	PC 2 (Executive)	PC 3 (Emotional)
Self-rated DAS Initiation	0.974^***^	0.321^***^	0.097
Self-rated DAS Executive	0.389^***^	0.984^***^	0.028
Self-rated DAS Emotional	0.178	0.090	0.652^***^
Self-rated DAS Total	0.810^***^	0.659^***^	0.294^***^

*In gray are those most applicable to each factor. Those considered relevant are shown in bold characters (≥ 0.30). PC, principal component*.

* *p < 0.05*;

** *p < 0.01*;

*** *p < 0.001*.

### Apathy Profile

There was no significant difference in age between the ALS patient subsample (Mean = 54.9, SD = 10.5) and controls (Mean = 53.2, SD = 10.5). Additionally, there was no significant difference in education level between the ALS patient subsample (36.7% university educated) and controls (44.9% university educated). The ALS patient subsample was perfectly matched to the control sample by sex.

**Figure 1:**
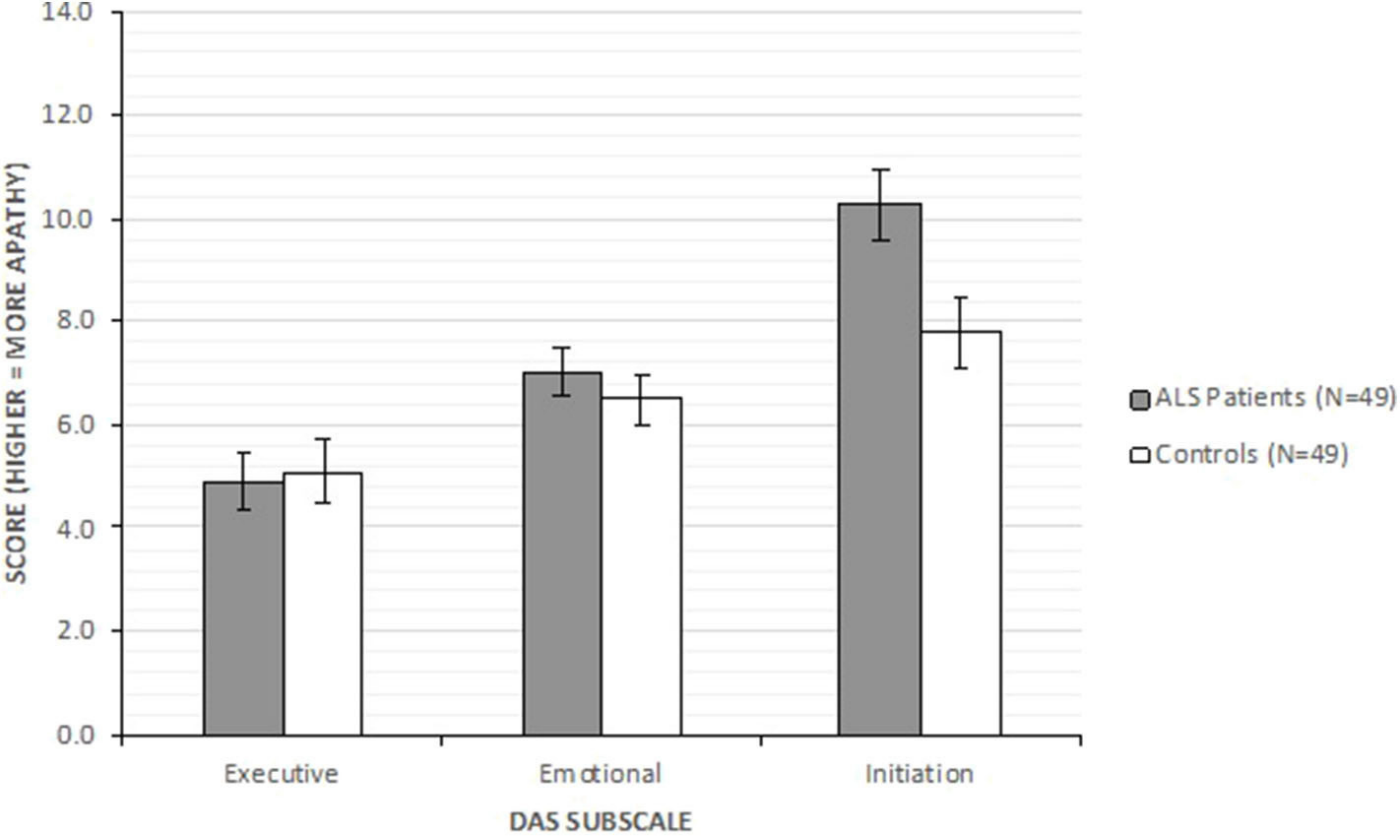
DAS subscales comparisons between ALS patient subsample (*n* = 49) and control (*n* = 49) groups.

The ALS subsample group was found to have significantly higher Initiation apathy than controls, with no significant differences were observed on Executive and Emotional apathy (see Figure 1).

The informant/carer-rated DAS Emotional apathy subscale (Mean = 9.22, SD = 4.04) scores was significantly higher than the patient self-rating (Mean = 7.12, SD = 3.16) scores [*t*_(66)_ = 3.268, *p* < 0.01]. No significant difference was found on DAS Executive apathy subscale scores between informant/caregiver-ratings (Mean = 5.61, SD = 4.29) and self-ratings (Mean = 5.70, SD = 4.45). There was no significant difference on the DAS Initiation apathy subscale scores between informant/caregiver-ratings (Mean = 10.01, SD = 6.19) and self-ratings (Mean = 9.37, SD = 4.78).

Normative data were used to suggest abnormal value thresholds for each subscale based on ≥2 SD over the mean (see Supplementary Table 1). Based on normative data, 12.2% of the ALS subsample group showed Initiation apathy, with 4.1% showing Executive apathy and 4.1% showing Emotional apathy. According to previously published cutoffs (16), 16.3% of the ALS subsample group showed Initiation apathy, with 4.1% showing Executive apathy and 2.0% showing Emotional apathy.

## Discussion

The Spanish DAS was found to be reliable and valid for the assessment of apathy subtypes. The substructure of the self-rated Spanish DAS was similar to that of the original DAS, providing support for it as a multidimensional measure. Further, exploration of the apathy profile in the Spanish ALS patients showed results similar to those of previous research (16, 17), with Initiation apathy (lack of motivation for self-generation of thoughts and/or actions) being characteristic and independent of physical disability.

Psychometrically, the self-rated Spanish DAS subscales all showed a good test-retest reliability, showing a relative stability of apathy subtypes in ALS patients. In terms of divergent validity, both the self-rated and informant/caregiver-rated DAS subscales were not associated with depression. In particular, the DAS Emotional apathy has previously been observed to have no association or a weak association with depression in ALS (16, 17), which has also been found for the Italian, Japanese, and French translations of the DAS (19–21). This adds support to the notion of Emotional apathy (i.e., emotional neutrality) and depression (i.e., emotionality) as disparate constructs. Moreover, the lack of association of Initiation and Executive apathy subscales against depression suggests that measurement of apathy with the Spanish DAS is independent of the confounding factor of depression. However, as with previous research, it is important to continue to measure apathy and depression together, as the constructs occur concurrently and have variable impacts (30). In terms of the intercorrelations of the Spanish DAS subscales, Executive and Initiation apathy seemed to be associated most consistently across the informant/caregiver-ratings and self-ratings. This is likely due to overlapping executive processes underlying motivation for initiation and planning/organizing/attention (12, 31).

While the self-rated Spanish DAS showed good internal consistency reliability, the informant/caregiver-rated version was observed to have higher internal consistency reliability. This is very much in line with previous research from ALS and dementia (16, 32), indicating the informant/caregiver-rated version is more psychometrically reliable. Furthermore, ALS patients with apathy/inertia (based on the semi-structured ECAS behavioral interview) had higher informant/caregiver-rated Spanish DAS scores, showing good convergent validity. However, no such difference was found for the self-rated Spanish DAS. Variable awareness of, or insight into, behavior problems and/or changes is characteristic of frontotemporal dementia associated with ALS (33), which may influence patients' self-ratings. As a result, the informant/caregiver-rated DAS was used to design the informant/caregiver-rated brief DAS (34, 35), a short and comprehensive apathy subtype measure with a supplemental awareness assessment. Future research should look to translate and utilize the brief DAS clinically to help detect motivational problems.

The substructure of the self-rated Spanish DAS has a high mapping percentage to the original DAS substructure (23) and to previous translations (19–21). The moderate to strong association between PC-derived subscores with relevant DAS subscores further provides support for the claim that the Spanish DAS is a valid instrument for multidimensional apathy assessment. The three-dimensional factor structure (akin to the original DAS) showed high mapping percentages relative to DAS Executive and Initiation subscales. The Emotional subscale was observed to map at a lower percentage; however, the items that did map were those included as a part of the b-DAS (items 5, 7, and 24) (34). The French translation of the DAS suggested that the Emotional apathy construct is more multifaceted, where further subcomponents of “individual” and “social” Emotional apathy might be of importance (20). Therefore, further transcultural research would be beneficial to understand how Emotional apathy is perceived and expressed in different countries.

Apathy profile exploration showed that Initiation apathy was observed to be characteristic in ALS when compared with matched controls, in line with previous findings (16, 17). Initiation apathy was also observed to be the higher in terms of DAS subscale cutoffs, albeit at a lower prevalence. The availability of culturally specific cutoffs will enable more specific exploration of apathy subtypes in the Spanish population. Emotional apathy was rated as higher by informant/caregivers, when compared to patient self-ratings, which could suggest lower awareness or insight of this apathy subtype. Research has further suggested that both Emotional apathy and lower awareness of Emotional apathy are observable in behavioral variant FTD (36); therefore, it would be of interest to explore apathy subtype profiles (specifically Emotional apathy) across ALS-frontotemporal spectrum disorders (ALS-FTSD) (33).

While the Spanish DAS has been shown to be valid and reliable, at the time of data collection there were no comparable valid “gold-standard” apathy measures available in Spanish as comparators, which prevented full convergent validity from being determined. This, however, did not limit the development of the original DAS and the French translation (20, 23). In the ECAS behavior interview the apathy/inertia domain was additionally utilized for validation of the informant/caregiver-rated Spanish DAS. Future research should explore the sensitivity, specificity, and predictive validity of the Spanish DAS to further determine its clinical utility. The convenience recruitment method utilized may also have biased the sample and, as with other apathy research, may have introduced a volunteerism bias. However, this is unlikely to impact the Spanish DAS validation results as previous DAS validation research has used similar recruitment methodology (16, 21, 35). Furthermore, there was an age difference between the ALS patient group and the control group. In terms of apathy profile results, previous subsampling methodology (16) was utilized to ensure the matching of ALS patients and controls with respect to age, sex, and education, for a more robust comparison.

In conclusion, the Spanish DAS is a psychometrically robust instrument for assessing multidimensional apathy, with three-dimensional factorial substructure. Further, Initiation apathy was found to be characteristic in Spanish ALS patients. Future research should look to further explore the clinical impact of apathy subtypes in different neurological, psychiatric, and neurodegenerative conditions.

## Data Availability Statement

The datasets generated for this study are available on request to the corresponding author.

## Ethics Statement

The studies involving human participants were reviewed and approved by the Institutional Review Board of The Institute University Hospital La Paz (IdiPAZ). The patients/participants provided their written informed consent to participate in this study.

## Author Contributions

All authors listed have made a substantial, direct and intellectual contribution to the work, and approved it for publication.

## Conflict of Interest

The authors declare that the research was conducted in the absence of any commercial or financial relationships that could be construed as a potential conflict of interest.

## References

[1] Goldstein L Abrahams S. Changes in cognition and behaviour in amyotrophic lateral sclerosis: nature of impairment and implications for assessment. Lancet Neurol. (2013) 12:368–80. 10.1016/S1474-4422(13)70026-7 23518330

[2] Grossman A Woolley-Levine S Bradley W Miller R. Detecting neurobehavioral changes in amyotrophic lateral sclerosis. Amyotroph Lateral Scler. (2007) 8:56–61. 10.1080/17482960601044106 17364437

[3] Witgert M Salamone A Strutt A Jawaid A Massman P Bradshaw M. Frontal-lobe mediated behavioral dysfunction in amyotrophic lateral sclerosis. Eur J Neurol. (2009) 17:103–10. 10.1111/j.1468-1331.2009.02801.x 19874396

[4] Lillo P Mioshi E Zoing M Kiernan M Hodges J. How common are behavioural changes in amyotrophic lateral sclerosis? Amyotroph Lateral Scler. (2010) 12:45–51. 10.3109/17482968.2010.520718 20849323

[5] Girardi A MacPherson S Abrahams S. Deficits in emotional and social cognition in amyotrophic lateral sclerosis. Neuropsychology. (2011) 25:53–65. 10.1037/a0020357 20919762

[6] Caga J Turner M R Hsieh S Ahmed RM Devenney E Ramsey E. Apathy is associated with poor prognosis in amyotrophic lateral sclerosis. Eur J Neurol. (2016) 23:891–7. 10.1111/ene.12959 26822417

[7] Grace J Malloy P Frontal Systems Behavior Scale. Lutz, FL: Assessment (2001). 10.1177/1073191113492845 23800608

[8] Wear H Wedderburn C Mioshi E Williams-Gray C Mason S Barker R. The Cambridge behavioural inventory revised. Dement Neuropsychol. (2008) 2:102–7. 10.1590/S1980-57642009DN20200005 29213551 PMC5619578

[9] Raaphorst J Beeldman E Schmand B Berkhout J Linssen W vanden Berg L. The ALS-FTD-Q: a new screening tool for behavioral disturbances in ALS. Neurology. (2012) 79:1377–83. 10.1212/WNL.0b013e31826c1aa1 22972650

[10] Mioshi E Hsieh S Caga J Ramsey E Chen K Lillo P. A novel tool to detect behavioural symptoms in ALS. Amyotroph Lateral Scler Frontotemporal Degener. (2014) 15:298–304. 10.3109/21678421.2014.896927 24863641

[11] Levy R Dubois B. Apathy and the functional anatomy of the prefrontal cortex–basal ganglia circuits. Cerebral Cortex. (2006) 16:916–28. 10.1093/cercor/bhj043 16207933

[12] Radakovic R Abrahams S Multidimensional apathy: evidence from neurodegenerative disease. Curr Opin Behav Sci. (2018) 22:42–9. 10.1016/j.cobeha.2017.12.022

[13] Robert P Onyike CU Leentjens AF Dujardin K Aalten P Starkstein S. Proposed diagnostic criteria for apathy in Alzheimer's disease and other neuropsychiatric disorders. Eur Psychiatry. (2009) 24:98–104. 10.1016/j.eurpsy.2008.09.001 19201579

[14] Robert P Lanctôt KL Agüera-Ortiz L Aalten P Bremond F Defrancesco M. Is it time to revise the diagnostic criteria for apathy in brain disorders? The 2018 international consensus group. Eur Psychiatry. (2018) 54:71–6. 10.1016/j.eurpsy.2018.07.008 30125783

[15] Manera V Abrahams S Agüera-Ortiz L Bremond F David R Fairchild K. Recommendations for the nonpharmacological treatment of apathy in brain disorders. Am J Geriatr Psychiatry. (2019) 28:410–20. 10.1016/j.jagp.2019.07.014 31495772

[16] Radakovic R Stephenson L Colville S Swingler R Chandran S Abrahams S. Multidimensional apathy in ALS: validation of the dimensional apathy scale. J Neurol Neurosurg Psychiatry. (2016) 87:663–9. 10.1136/jnnp-2015-310772 26203157

[17] Santangelo G Siciliano M Trojano L Femiano C Monsurrò MR Tedeschi G. Apathy in amyotrophic lateral sclerosis: insights from dimensional apathy scale. Amyotroph Lateral Scler Frontotemporal Degener. (2017) 18:434–42. 10.1080/21678421.2017.1313865 28431489

[18] Radakovic R Stephenson L Newton J Crockford C Swingler R Chandran S. Multidimensional apathy and executive dysfunction in amyotrophic lateral sclerosis. Cortex. (2017) 94:142–51. 10.1016/j.cortex.2017.06.023 28759804

[19] Santangelo G Raimo S Siciliano M D'Iorio A Piscopo F Cuoco S. Assessment of apathy independent of physical disability: validation of the dimensional apathy scale in Italian healthy sample. Neurol Sci. (2016) 38:303–9. 10.1007/s10072-016-2766-8 27844173

[20] M'Barek L Radakovic R Noquet M Laurent A Allain P Different aspects of emotional processes in apathy: application of the French translated dimensional apathy scale. Current Psychology. (2018) 39:564–70. 10.1007/s12144-017-9775-5

[21] Kawagoe T Onoda K Yamaguchi S Radakovic R. Developing and validating the Japanese version of Dimensional Apathy Scale (J-DAS). Psychiatry Clin Neurosci. (2020) 74:411–2. 10.1111/pcn.13009 32304331 PMC7383736

[22] Brooks BR Miller RG Swash M Munsat TL. El Escorial revisited: revised criteria for the diagnosis of amyotrophic lateral sclerosis. Amyotroph Lateral Scler Other Motor Neuron Disord. (2000) 1:293–9. 10.1080/146608200300079536 11464847

[23] Radakovic R Abrahams S. Developing a new apathy measurement scale: dimensional apathy scale. Psychiatry Res. (2014) 219:658–63. 10.1016/j.psychres.2014.06.010 24972546

[24] Yesavage J Brink T Rose T Lum O Huang V Adey M. Development and validation of a geriatric depression screening scale: a preliminary report. J Psychiatr Res. (1982) 17:37–49. 10.1016/0022-3956(82)90033-4 7183759

[25] Martínez J Onís MC Dueñas R Colomer A Luque R Taberna C Versión española del cuestionario de Yesavage abreviado para el despistaje de depresión en mayores de 65 años. Medifam. (2002) 12:620–30. 10.4321/S1131-57682002001000003

[26] Abrahams S Newton J Niven E Foley J Bak TH. Screening for cognition and behaviour changes in ALS. Amyotroph Lateral Scler Frontotemporal Degener. (2014) 15:9–14. 10.3109/21678421.2013.805784 23781974

[27] Mora JS Salas T Fernández MC Rodríguez-Castillo V Marín S Chaverri D. Spanish adaptation of the edinburgh cognitive and behavioral amyotrophic lateral sclerosis screen (ECAS). Amyotroph Lateral Scler Frontotemporal Degener. (2018) 19:74–9. 10.1080/21678421.2017.1406952 29212378

[28] Cedarbaum JM Stambler N Malta E Fuller C Hilt D Thurmond B. The ALSFRS-R: a revised ALS functional rating scale that incorporates assessments of respiratory function. J Neurol Sci. (1999) 169:13–21. 10.1016/S0022-510X(99)00210-5 10540002

[29] Campos TS Rodríguez-Santos F Esteban J Vázquez PC Pardina JSM Carmona AC Adaptación Española de la Escala revisada de Valoración Funcional de la Esclerosis Lateral Amiotrófica (ALSFRS-R). Available online at: https://www.fundela.es/FilesRepo/L/9/C/W/pQqocrYNZt-adaptacionalsfrsr.pdf

[30] Tagariello P Girardi P Amore M Depression and apathy in dementia: same syndrome or different constructs? A critical review. Arch Gerontol Geriatr. (2009) 49:246–9. 10.1016/j.archger.2008.09.002 19022508

[31] Stuss DT. Functions of the frontal lobes: relation to executive functions. J Int Neuropsychol Soc. (2011) 17:759–65. 10.1017/S1355617711000695 21729406

[32] Radakovic R Starr JM Abrahams S. A novel assessment and profiling of multidimensional apathy in Alzheimer's disease. J Alzheimer's Dis. (2017) 60:57–67. 10.3233/JAD-170292 28759970

[33] Strong MJ Abrahams S Goldstein LH Woolley S Mclaughlin P Snowden J. Amyotrophic lateral sclerosis-frontotemporal spectrum disorder (ALS-FTSD): revised diagnostic criteria. Amyotroph Lateral Scler Frontotemporal Degener. (2017) 18:153–74. 10.1080/21678421.2016.1267768 28054827 PMC7409990

[34] Radakovic R McGrory S Chandran S Swingler R Pal S Stephenson L. The brief dimensional apathy scale: a short clinical assessment of apathy. Clin Neuropsychol. (2019) 34:423–35. 10.1080/13854046.2019.1621382 31154933

[35] Radakovic R Gray D Dudley K Mioshi E Dick D Melchiorre G. Reliability and validity of the brief dimensional apathy scale. Arch Clin Neuropsychol. (2020) 35:539–44. 10.1093/arclin/acaa002 32045001

[36] Radakovic R Colville S Cranley D Starr JM Pal S Abrahams S. Multidimensional apathy in behavioural variant frontotemporal dementia, primary progressive aphasia and Alzheimer's disease. J Geriatr Psychiatry Neurol. (2020) 1–8. 10.1177/0891988720924716. [Epub ahead of print]. 32410488 PMC8326892

