# Supplementary material for: Spanish Adaptation of the Dimensional Apathy Scale (DAS) in Amyotrophic Lateral Sclerosis
Source: Front Neurol. 2020 Oct 6;11:562837. doi: 10.3389/fneur.2020.562837 (PMC7573163; doi:10.3389/fneur.2020.562837)
Supplement: Supplementary file 1 [file Data_Sheet_1.PDF]

DAS Escala para la Medición de la Apatía (Autoevaluación) Paciente:

Edad……….. Sexo………….. Estado Civil……………………..

Años de Educación…………………………………………

Elija la respuesta que mejor describa cómo se ha **sentido, comportado o pensado**, durante el último mes. (Marque con un círculo la respuesta más apropiada)

1. Necesito un cierto estímulo para empezar
   - ◊ Casi siempre
   - ◊ A menudo
   - ◊ A veces
   - ◊ Casi nunca

2. Me pongo en contacto con mis amigos
   - ◊ Casi siempre
   - ◊ A menudo
   - ◊ A veces
   - ◊ Casi nunca

3. Expreso mis emociones
   - ◊ Casi siempre
   - ◊ A menudo
   - ◊ A veces
   - ◊ Casi nunca

4. Se me ocurren cosas nuevas que hacer a lo largo del día
   - ◊ Casi siempre
   - ◊ A menudo
   - ◊ A veces
   - ◊ Casi nunca

5. Me preocupa cómo se siente mi familia
   - ◊ Casi siempre
   - ◊ A menudo
   - ◊ A veces
   - ◊ Casi nunca

6. Me quedo mirando a las musarañas
   - ◊ Casi siempre
   - ◊ A menudo
   - ◊ A veces
   - ◊ Casi nunca

7. Antes de hacer algo pienso en cómo le sentará a los demás
   - ◊ Casi siempre
   - ◊ A menudo
   - ◊ A veces
   - ◊ Casi nunca

8. Planifico con anterioridad mis actividades diarias
   - ◊ Casi siempre
   - ◊ A menudo
   - ◊ A veces
   - ◊ Casi nunca

9. Me siento mal cuando me dan malas noticias
   - ◊ Casi siempre
   - ◊ A menudo
   - ◊ A veces
   - ◊ Casi nunca

10. Puedo centrarme en una tarea hasta haberla acabado
    - ◊ Casi siempre
    - ◊ A menudo
    - ◊ A veces
    - ◊ Casi nunca

11. Me falta motivación
    - ◊ Casi siempre
    - ◊ A menudo
    - ◊ A veces
    - ◊ Casi nunca

12. Me cuesta tener empatía con la gente
    - ◊ Casi siempre
    - ◊ A menudo
    - ◊ A veces
    - ◊ Casi nunca

© Copyright, The University Court of the University of Edinburgh and R. Radakovic, 2011 – 2014. All Right Reserved. The authors being S. Abrahams & R. Radakovic

13. Me marco objetivos
    - ◊ Casi siempre
    - ◊ A menudo
    - ◊ A veces
    - ◊ Casi nunca

14. Pruebo cosas nuevas
    - ◊ Casi siempre
    - ◊ A menudo
    - ◊ A veces
    - ◊ Casi nunca

15. No me importa lo que piensen los demás sobre mi comportamiento
    - ◊ Casi siempre
    - ◊ A menudo
    - ◊ A veces
    - ◊ Casi nunca

16. Realizo los asuntos que he pensado a lo largo del día
    - ◊ Casi siempre
    - ◊ A menudo
    - ◊ A veces
    - ◊ Casi nunca

17. Me cuesta decidir, cómo debo de actuar en una tarea que requiere esfuerzo
    - ◊ Casi siempre
    - ◊ A menudo
    - ◊ A veces
    - ◊ Casi nunca

18. Me mantengo ocupado
    - ◊ Casi siempre
    - ◊ A menudo
    - ◊ A veces
    - ◊ Casi nunca

19. Me confundo con facilidad cuando hago varias cosas a la vez
    - ◊ Casi siempre
    - ◊ A menudo
    - ◊ A veces
    - ◊ Casi nunca

20. Me emociono con facilidad cuando veo algo alegre o triste en la televisión
    - ◊ Casi siempre
    - ◊ A menudo
    - ◊ A veces
    - ◊ Casi nunca

21. Me cuesta centrarme en las cosas
    - ◊ Casi siempre
    - ◊ A menudo
    - ◊ A veces
    - ◊ Casi nunca

22. Soy espontáneo
    - ◊ Casi siempre
    - ◊ A menudo
    - ◊ A veces
    - ◊ Casi nunca

23. Me distraigo con facilidad
    - ◊ Casi siempre
    - ◊ A menudo
    - ◊ A veces
    - ◊ Casi nunca

24. No me importa lo que ocurre a mi alrededor
    - ◊ Casi siempre
    - ◊ A menudo
    - ◊ A veces
    - ◊ Casi nunca

© Copyright, The University Court of the University of Edinburgh and R. Radakovic, 2011 – 2014. All Right Reserved. The authors being S. Abrahams & R. Radakovic
