# Supplementary material for: Spanish Adaptation of the Dimensional Apathy Scale (DAS) in Amyotrophic Lateral Sclerosis
Source: Front Neurol. 2020 Oct 6;11:562837. doi: 10.3389/fneur.2020.562837 (PMC7573163; doi:10.3389/fneur.2020.562837)
Supplement: Supplementary file 2 [file Data_Sheet_2.PDF]

SELF - DAS (ESCALA DE MEDICIÓN DE LA APATÍA)

Instrucciones de Valoración

Utilice las instrucciones de valoración incluidas a continuación, sume los resultados totales para cada una de las distintas escalas.

Instrucciones de Valoración

**Elemento con valoración positiva +**		**Elemento con valoración negativa -**	
◊ Casi siempre	**0**	◊ Casi siempre	**3**
◊ A menudo	**1**	◊ A menudo	**2**
◊ A veces	**2**	◊ A veces	**1**
◊ Casi nunca	**3**	◊ Casi nunca	**0**

Tabla de puntuación

Escala Funciones Ejecutivas

Elemento	Puntos
1	
6	
10+	
11	
17	
19	
21	
23	

Total:

Escala Emocional

Elemento	Puntos
3+	
5+	
7+	
9+	
12	
15	
20+	
24	

Total:

Escala inicio Cognitivo/Conductual

Elemento	Puntos
2+	
4+	
8+	
13+	
14+	
16+	
18+	
22+	

Total:

© Copyright, The University Court of the University of Edinburgh and R. Radakovic, 2011 – 2014.
All Right Reserved. The authors being S. Abrahams & R. Radakovic
