# Supplementary material for: Spanish Adaptation of the Dimensional Apathy Scale (DAS) in Amyotrophic Lateral Sclerosis
Source: Front Neurol. 2020 Oct 6;11:562837. doi: 10.3389/fneur.2020.562837 (PMC7573163; doi:10.3389/fneur.2020.562837)
Supplement: Supplementary file 3 [file Data_Sheet_3.PDF]

Relación con el paciente……………………………………………

Elija la respuesta que mejor describa, según sus observaciones, cómo se ha **sentido, comportado o cómo ha razonado** el paciente, basándose en su habitualidad durante el último mes (Marque con un círculo la respuesta más adecuada)

1. El/ella necesita un cierto estímulo para empezar
   - ◊ Casi siempre
   - ◊ A menudo
   - ◊ A veces
   - ◊ Casi nunca

2. El/ella mantiene el contacto con sus amistades
   - ◊ Casi siempre
   - ◊ A menudo
   - ◊ A veces
   - ◊ Casi nunca

3. El/ella expresa sus emociones
   - ◊ Casi siempre
   - ◊ A menudo
   - ◊ A veces
   - ◊ Casi nunca

4. A El/ella se le ocurren cosas nuevas que hacer a lo largo del día
   - ◊ Casi siempre
   - ◊ A menudo
   - ◊ A veces
   - ◊ Casi nunca

5. El/ella se preocupa por los sentimientos de sus familiares
   - ◊ Casi siempre
   - ◊ A menudo
   - ◊ A veces
   - ◊ Casi nunca

6. El/ella se queda mirando a las musarañas
   - ◊ Casi siempre
   - ◊ A menudo
   - ◊ A veces
   - ◊ Casi nunca

7. A El/ella antes de hacer algo piensa en cómo le sentará a los demás
   - ◊ Casi siempre
   - ◊ A menudo
   - ◊ A veces
   - ◊ Casi nunca

8. El/ella planifica con anterioridad sus actividades diarias
   - ◊ Casi siempre
   - ◊ A menudo
   - ◊ A veces
   - ◊ Casi nunca

9. El/ella se siente mal cuando le dan malas noticias
   - ◊ Casi siempre
   - ◊ A menudo
   - ◊ A veces
   - ◊ Casi nunca

10. El/ella puede centrarse en una tarea hasta haberla acabado
    - ◊ Casi siempre
    - ◊ A menudo
    - ◊ A veces
    - ◊ Casi nunca

11. El/ella le falta motivación
    - ◊ Casi siempre
    - ◊ A menudo
    - ◊ A veces
    - ◊ Casi nunca

12. El/ella le cuesta tener empatía con la gente
    - ◊ Casi siempre
    - ◊ A menudo
    - ◊ A veces
    - ◊ Casi nunca

© Copyright, The University Court of the University of Edinburgh and R. Radakovic, 2011 – 2015. All Right Reserved. The authors being S. Abrahams & R. Radakovic

13. El/ella se pone metas
    - ◊ Casi siempre
    - ◊ A menudo
    - ◊ A veces
    - ◊ Casi nunca

14. El/ella prueba cosas nuevas
    - ◊ Casi siempre
    - ◊ A menudo
    - ◊ A veces
    - ◊ Casi nunca

15. A El/ella no le importa lo que piensen los demás sobre su comportamiento
    - ◊ Casi siempre
    - ◊ A menudo
    - ◊ A veces
    - ◊ Casi nunca

16. El/ella actúa sobre los asuntos que ha pensado a lo largo del día
    - ◊ Casi siempre
    - ◊ A menudo
    - ◊ A veces
    - ◊ Casi nunca

17. El/ella cuando realiza una tarea que requiere esfuerzo, le cuesta decidir qué debe hacer
    - ◊ Casi siempre
    - ◊ A menudo
    - ◊ A veces
    - ◊ Casi nunca

18. El/ella se mantiene ocupado
    - ◊ Casi siempre
    - ◊ A menudo
    - ◊ A veces
    - ◊ Casi nunca

19. El/ella se confunde con facilidad cuando hace varias cosas a la vez
    - ◊ Casi siempre
    - ◊ A menudo
    - ◊ A veces
    - ◊ Casi nunca

20. El/ella se emociona con facilidad cuando ve algo alegre o triste en la televisión
    - ◊ Casi siempre
    - ◊ A menudo
    - ◊ A veces
    - ◊ Casi nunca

21. A El/ella le cuesta centrarse en las cosas
    - ◊ Casi siempre
    - ◊ A menudo
    - ◊ A veces
    - ◊ Casi nunca

22. El/ella es espontáneo/a
    - ◊ Casi siempre
    - ◊ A menudo
    - ◊ A veces
    - ◊ Casi nunca

23. El/ella se distrae con facilidad
    - ◊ Casi siempre
    - ◊ A menudo
    - ◊ A veces
    - ◊ Casi nunca

24. El/ella no le importa lo que ocurre a su alrededor
    - ◊ Casi siempre
    - ◊ A menudo
    - ◊ A veces
    - ◊ Casi nunca

© Copyright, The University Court of the University of Edinburgh and R. Radakovic, 2011 – 2015. All Right Reserved. The authors being S. Abrahams & R. Radakovic

DAS (ESCALA DIMENSIONAL DE LA APATÍA) - CUIDADORES

Instrucciones para la puntuación

Utilizando las instrucciones para la puntuación, a continuación, sume las puntuaciones totales de cada una de las subescalas.

Instrucciones para la puntuación

**Puntuación positiva +**		**Puntuación negativa**	
◊ Casi siempre	**0**	◊ Casi siempre	**3**
◊ A menudo	**1**	◊ A menudo	**2**
◊ A veces	**2**	◊ A veces	**1**
◊ Casi nunca	**3**	◊ Casi nunca	**0**

Tabla de puntuación

Subescala Ejecutiva

Elemento	Puntuación
1	
6	
10+	
11	
17	
19	
21	
23	

Total:

Subescala Emocional

Elemento	Puntuación
3+	
5+	
7+	
9+	
12	
15	
20+	
24	

Total:

Comportamiento/Subescala de Iniciación Cognitiva

Elemento	Puntuación
2+	
4+	
8+	
13+	
14+	
16+	
18+	
22+	

Total:

© Copyright, The University Court of the University of Edinburgh and R. Radakovic, 2011 – 2015. All Right Reserved. The authors being S. Abrahams & R. Radakovic
