# Supplementary material for: Spanish Adaptation of the Dimensional Apathy Scale (DAS) in Amyotrophic Lateral Sclerosis
Source: Front Neurol. 2020 Oct 6;11:562837. doi: 10.3389/fneur.2020.562837 (PMC7573163; doi:10.3389/fneur.2020.562837)
Supplement: Supplementary file 4 [file Table_1.DOCX]

**Supplementary Table 1.** DAS Spanish normative data (N=49) and cut-offs.

|  | Mean (SD) | Range | Cut-off |
| --- | --- | --- | --- |
| DAS Executive | 5.10 (4.28) | 0-16 | ≥ 14 |
| DAS Emotional | 6.49 (3.32) | 0-14 | ≥ 13 |
| DAS Initiation | 7.80 (4.67) | 0-20 | ≥ 17 |
| DAS Total | 19.39 (9.31) | 4-47 | ≥ 38 |
